# Quality Assessment of Fish Oil Obtained after Enzymatic Hydrolysis of a Mixture of Rainbow Trout (*Oncorhynchus mykiss)* and Atlantic Salmon (*Salmo salar*) Rest Raw Material Pretreated by High Pressure

**DOI:** 10.3390/md22060261

**Published:** 2024-06-05

**Authors:** Elissavet Kotsoni, Egidijus Daukšas, Grete Hansen Aas, Turid Rustad, Brijesh K. Tiwari, Janna Cropotova

**Affiliations:** 1 Department of Biological Sciences Ålesund, Norwegian University of Science and Technology, 6009 Ålesund, Norway; egidijus.dauksas@ntnu.no (E.D.); graa@ntnu.no (G.H.A.); janna.cropotova@ntnu.no (J.C.); 2 Department of Biotechnology and Food Science, Norwegian University of Science and Technology, 7034 Trondheim, Norway; turid.rustad@ntnu.no; 3 Food Chemistry and Technology Department, Teagasc Food Research Centre, Ashtown, D15 DY05 Dublin, Ireland; brijesh.tiwari@teagasc.ie

**Keywords:** novel technologies, utilization of fish side streams, fatty acids, lipid oxidation, storage stability

## Abstract

Utilization of fish rest raw material for fish oil extraction has received interest with the increasing demand for sustainable food sources. Enzymatic hydrolysis is an efficient method for the extraction of value-added compounds, but its effectiveness may be enhanced by high-pressure processing (HPP). However, HPP can induce lipid oxidation, affecting the quality of the oil. This study aimed to evaluate the quality of fish oil obtained after enzymatic hydrolysis of a mixture of rainbow trout (*Oncorhynchus mykiss*) and Atlantic salmon (*Salmo salar*) rest raw material pretreated by HPP. Six pretreatments were tested prior to enzymatic hydrolysis; 200 MPa × 4 min, 200 MPa × 8 min, 400 MPa × 4 min, 400 MPa × 8 min, 600 MPa × 4 min, and 600 MPa × 8 min. The oil samples were analyzed for lipid oxidation parameters, free fatty acid content, fatty acid composition, and color changes over 8 weeks. The results confirmed that HPP may induce lipid oxidation and revealed significant influence of HPP parameters on lipid oxidation, with higher pressures leading to increased oxidation. Fatty acid composition varied among samples, but it was not substantially affected by HPP.

## 1. Introduction

In recent years, there has been a noticeable increase in the demand for food sources that are both nutritionally rich and sustainable [1]. This trend is largely driven by the rapidly expanding global population, which is projected to reach 9.8 billion by 2050, as reported by the United Nations [2]. To meet this growing demand, the exploitation of fish rest raw material, including trimmings, frames, bones, viscera, heads, skin, and belly flaps—which may constitute up to 70% of the total processed fish—has emerged as a sustainable solution. Traditionally, this material has been either discarded as waste from processing or utilized in producing animal feed and fertilizers [3,4].

Fish oil is rich in n-3 polyunsaturated fatty acids (PUFAs), especially eicosapentaenoic acid (EPA) and docosahexaenoic acid (DHA). These fatty acids have been shown to demonstrate positive effects on human health by reducing the risk associated with hypertension and cardiovascular diseases, as well as autoimmune and inflammatory disorders [5,6]. Fish rest raw material offers a good potential for producing fish oil, particularly for applications within the pharmaceutical and nutraceutical industries [7,8]. The extraction of fish oil from fish rest raw material not only supports sustainability but also aligns with the principles of the circular economy by utilizing their high nutrient and bioactive compound content efficiently [9].

Enzymatic hydrolysis is an effective method for extracting oil from fish rest raw material, mainly using exogenous enzymes to facilitate the process [10,11]. Despite the cost of the enzymes, the long processing time, and the high temperatures required for the inactivation of the enzymes [12,13], enzymatic hydrolysis is valued for its ease of control and high reproducibility compared to alternative methods like heat extraction, hydraulic pressing, and supercritical fluid extraction [14,15,16]. One of the advantages of enzymatic hydrolysis is its operation at low temperatures, resulting in a higher quality and better recovery rate of the extracted oil compared to oils obtained through alternative methods. However, various factors such as hydrolysis conditions, fish species, and the type of fish rest raw material utilized can significantly impact the obtained oil yield [17,18]. 

Studies have shown that the implementation of innovative non-thermal technologies, such as high-pressure processing (HPP), prior to enzymatic hydrolysis can enhance the recovery of value-added compounds from fish rest raw material, such as lipids and proteins [19,20]. HPP may improve oil extraction by disrupting cell membranes and facilitating enzymatic activity. The quantity of oil released is often linked to the degree of protein denaturation, influenced by the conditions of the applied HPP [20]. However, in our earlier publication the oil yield was mostly unaffected by HPP [21]. This process can induce lipid oxidation, particularly affecting PUFAs abundant in fish oil, which may alter the sensory and nutritional properties of the oil [22]. The mechanism of lipid oxidation under HPP involves the formation of free radicals during the pressurization phase, especially at pressures above 300 MPa. Lipid oxidation is triggered by the release of both pro-oxidants, following membrane destruction, and free metals from pressure-induced protein denaturation [22]. These radicals can react with the unsaturated bonds of fatty acids like EPA and DHA, initiating a chain reaction that leads to the formation of lipid hydroperoxides and their subsequent breakdown products. Hence, the addition of antioxidants into the fish rest raw material can scavenge free radicals and stabilize the lipids during storage [23,24]. Furthermore, HPP may promote the release of natural antioxidants like astaxanthin, which are abundant in salmonids [25]. 

The objective of the current study was to assess the quality of fish oil extracted through enzymatic hydrolysis from a mixture of rainbow trout and Atlantic salmon rest raw material pretreated by high pressure (HP). Specifically, the research focused on investigating the impact of various HPP conditions on lipid oxidation parameters, free fatty acid content, fatty acid composition, and color stability of the fish oil throughout storage. One hypothesis was that higher pressure and longer time would lead to increased lipid oxidation. Further, the second hypothesis was that the added antioxidants would limit lipid oxidation.

## 2. Results and Discussion

### 2.1. Lipid Oxidation Parameters

**Figure 1:**
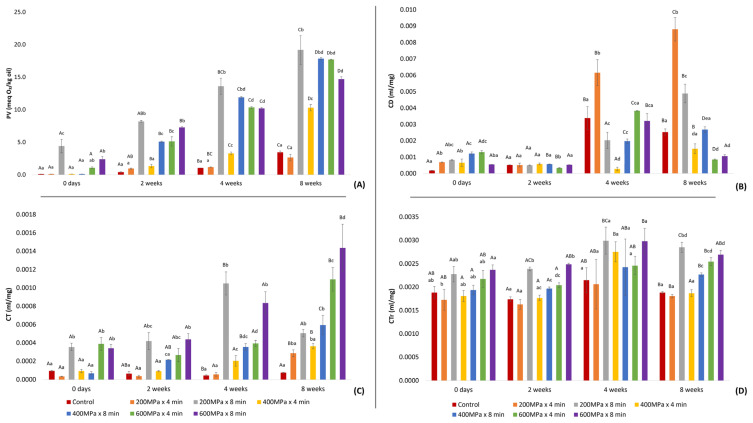
Determination of primary lipid oxidation products in oil extracted after enzymatic hydrolysis on samples consisting of a mixture of rainbow trout and Atlantic salmon rest raw material subjected to HPP and on the control during storage: (**A**) PV, expressed as meq O_2_/kg oil (mean ± SD, n = 2); (**B**) CD, expressed as ml/mg oil (mean ± SD, n = 5); (**C**) CT, expressed as mg/mL oil (mean ± SD, n = 5); (**D**) CTr, expressed as mg/mL oil (mean ± SD, n = 5). In each treatment group, bars marked with distinct uppercase letters denote a significant difference. Across various treatment groups, bars labeled with different lowercase letters indicate significant differences.

A significant difference in PV was observed among fish oil samples subjected to HP pretreatment and control throughout the storage period (Figure 1A). Immediately after production (0 days), oil from samples pretreated at 200 MPa × 4 min, 400 MPa × 8 min, 400 MPa × 4 min, and the control exhibited a PV below 0.1 meq O_2_/kg oil, whereas the oil from the sample subjected to pretreatment at 200 Mpa × 8 min demonstrated the highest PV. This result could suggest that the propagation stage of lipid oxidation in this specific sample initiated earlier in the process, influencing the oxidation rate during storage. These findings are in contrast with those reported by Zhang, Sun, Liu, Wei, Xia, Ji, Deng and Hao [20], where the PV of the oil obtained after enzymatic hydrolysis of tuna heads pretreated by ultra-high pressure was 11 meq O_2_/kg oil. This difference could possibly be attributed to the addition of natural antioxidants and nitrogen in the hydrolysis vessel to avoid contact between oxygen and the raw material during enzymatic hydrolysis in the current study, and therefore, a delay in the oxidation process. Additionally, the presence of astaxanthin, whose release may be enhanced by HPP, could have contributed to antioxidant activity, as indicated by previous studies [19,25]. Despite this, all treatments displayed an increase in the PV over time; the rate and extent of this increase varied among the oil samples. This suggests that while the addition of natural antioxidants may have initially provided some protection against lipid oxidation, it was not sufficient to ensure oxidative stability throughout the storage period [23]. Notably, samples pretreated at moderate and high pressures, particularly oil from samples pretreated at 400 MPa × 8 min, 600 MPa × 4 min, and 600 MPa × 8 min, exhibited a more pronounced increase compared to the rest of the samples as time elapsed. Specifically, after 4 weeks of storage, these specific samples displayed PVs higher than 5 meq O_2_/kg oil, which is considered the acceptable level for fish oil directed to human consumption [26]. Conversely, oil obtained from the sample pretreated at 200 MPa × 4 min did not significantly differ from the PV of the control. After 8 weeks of storage, the PV remained below 5 meq O_2_/kg oil for both this sample and the control. Although the former (200 MPa × 4 min) displayed a lower PV compared to the control, this difference was not statistically significant. Additionally, a correlation was observed between HP pretreatment and PV at 2 and 4 weeks after production (r = 0.843 and r = 0.830, respectively), highlighting the effect of HPP on the oxidative stability of fish oil.

The analysis of conjugated systems revealed significant variations in the levels of CD, CT, and CTr in the oil samples under various HPP conditions throughout the storage period. Immediately after production and after 2 weeks of storage, all oil samples exhibited low CD levels (Figure 1B). Nevertheless, after 4 weeks of storage, the values of CD demonstrated a significant increase in the majority of oil samples. The most pronounced CD increase was detected in the oil obtained from the sample subjected to 200 MPa × 4 min, followed by the control oil and those from samples pretreated at 600 MPa × 4 min and 600 MPa × 8 min; however, the variation in CD levels among the latter three samples was not significant. With respect to CT (Figure 1C), the oils from samples pretreated at 200 MPa × 4 min and 400 MPa × 4 min followed a trend similar to the control, while the remaining oil samples displayed significantly higher values across the storage period. A correlation between the pretreatment conditions and CT emerged only after 8 weeks of storage (r = 0.894), indicating the significant impact of pressure on the observed increased CT values over a prolonged storage period. As illustrated in Figure 1D, CTr demonstrated a comparable pattern to that of the CT values. This observation is supported by the significant correlation between CT and CTr detected immediately after production (r = 0.925) and during the storage period at 2 and 4 weeks (r = 0.993 and r = 0.863, respectively).

**Figure 2:**
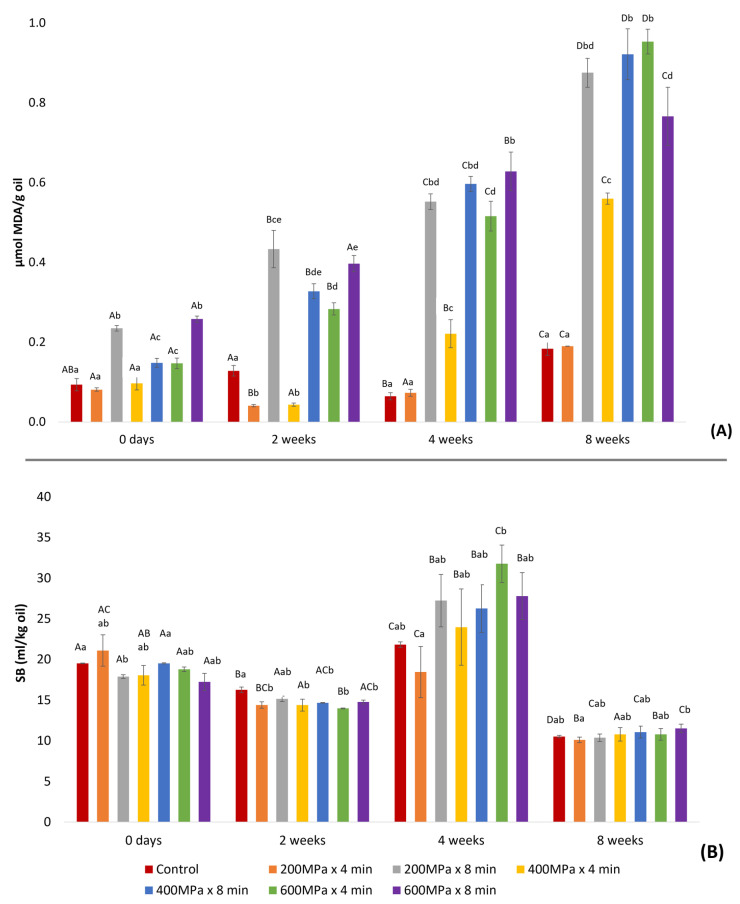
Measurement of secondary lipid oxidation products in oil extracted after enzymatic hydrolysis on samples consisting of a mixture of rainbow trout and Atlantic salmon rest raw material subjected to HPP and on the control during storage: (**A**) TBARSs, expressed as μmol MDA/g oil (mean ± SD, n = 2); (**B**) SBs, expressed as ml/kg oil (mean ± SD, n = 3). In each treatment group, bars marked with distinct uppercase letters denote a significant difference. Across various treatment groups, bars labeled with different lowercase letters indicate significant differences.

The various applied HPP conditions significantly influenced the TBARS values, resembling the effect observed on PV across all oil samples during storage (Figure 2A).

This association is highlighted by a significant correlation observed between PV and TBARSs throughout the whole storage period, indicating the accumulation of secondary oxidation products due to the degradation of lipid hydroperoxides [27]. Specifically, at 0 days after production the correlation coefficient was r = 0.856. Subsequently, after 2, 4, and 8 weeks of storage, the correlation coefficients were r = 0.961, r = 0.961, and r = 0.990, respectively. Oil derived from samples pretreated at 400 MPa × 8 min, 600 MPa × 4 min, and 600 MPa × 8 min exhibited a more noticeable increase in TBARS levels as storage progressed. Oil derived from samples pretreated at 400 MPa × 8 min, 600 MPa × 4 min, and 600 MPa × 8 min exhibited a more noticeable increase in TBARS levels as storage progressed. In contrast, oil from the sample pretreated at 200 MPa × 4 min displayed TBARS values either lower than or comparable to those of the control oil. However, the difference in TBARSs between the oil from the sample pretreated at 200 MPa × 4 min and the control was not significant. These findings suggest that the oils obtained from the sample pretreated at 200 MPa × 4 min and the control may exhibit greater stability during storage. Moreover, a correlation was found between pretreatment and TBARS values at 4 and 8 weeks after production (r = 0.893 and r = 0.823, respectively), suggesting that HPP may affect the development of lipid oxidation over time.

It is possible that secondary lipid oxidation products began to form during the early stages of raw material processing, prior to HP pretreatment and enzymatic hydrolysis, and were subsequently extracted into the oil. This hypothesis is supported by the TBARS values detected in the oil at 0 days, suggesting that the generated secondary lipid oxidation products (aldehydes) might have reacted with side-chain amino groups present in the raw material, leading to SB formation [28]. After 4 weeks of storage, an increase in the amount of SBs was observed in all samples, likely due to the increase in the generation of secondary lipid oxidation products involved in SB formation. This is further supported by the significant correlation (r = 0.835) found between SBs and TBARSs after 4 weeks of storage. The subsequent decrease in SB levels after 8 weeks of storage may be explained by the formation of complexes between SBs and other compounds, making them less detectable [29].

### 2.2. Determination of FFAs

**Figure 3:**
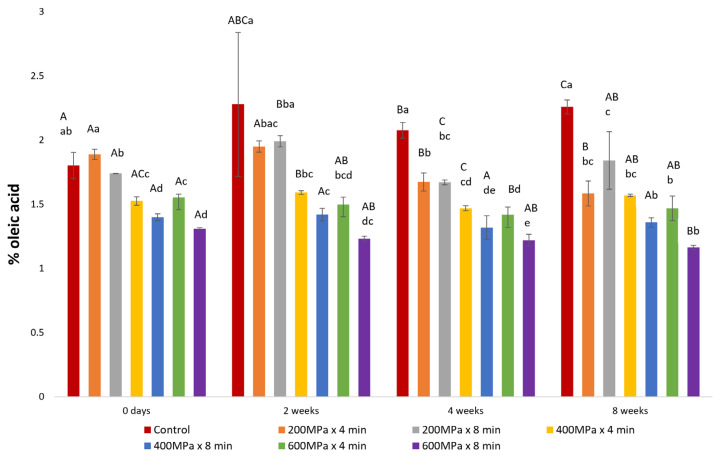
Illustration of change in FFA content over storage period in oil extracted after enzymatic hydrolysis on samples consisting of a mixture of rainbow trout and Atlantic salmon rest raw material subjected to HPP and on the control, expressed as g oleic acid/100 g oil (mean ± SD, n = 3). In each treatment group, bars marked with distinct uppercase letters denote a significant difference. Across various treatment groups, bars labeled with different lowercase letters indicate significant differences.

The HPP conditions, particularly the applied pressure, exerted a significant influence on the FFA content within the extracted oil fractions (Figure 3). Throughout the storage period, oil derived from samples subjected to low pressure (200 MPa) and the control exhibited significantly higher FFA levels compared to oil derived from samples pretreated at moderate (400 MPa) and high pressures (600 MPa). Furthermore, oil from samples pretreated with HP for 8 min demonstrated lower FFA levels compared to those pretreated for 4 min at equivalent pressure levels, indicating that prolonged HP pretreatment could reduce the FFA content. This reduction may be attributed to the inactivation of lipases under pressure in the initial raw material prior to enzymatic hydrolysis [30]. Application of higher pressure for a longer duration can lead to increased inactivation of lipases, and therefore, a lower amount of generation of FFAs, which are susceptible to oxidation [31,32]. The FFA levels in oil obtained from the control and samples pretreated at 200 MPa × 8 min, 400 MPa × 8 min, and 600 MPa × 4 min remained stable during storage. Nonetheless, a fluctuation in FFA content was observed in oils from samples pretreated at 200 MPa × 4 min, 400 MPa × 4 min, and 600 MPa × 8 min. These observations could suggest that specific HPP conditions may influence the rate of lipid oxidation over time. This hypothesis could be supported by the significant correlation revealed between FFA content and CD (r = 0.990) in oil extracted from the sample pretreated at 200 MPa × 4 min, the correlation between FFA content and CTr (r = 0.869) in oil extracted from the sample pretreated at 400 MPa × 4 min, and the significant correlation between FFA content and PV and TBARSs (r = 0.985 and r = 0.934, respectively) in oil extracted from the sample pretreated at 600 MPa × 8 min. However, in all cases, the FFA content remained below 3% of oleic acid, aligning with the recommendation for high-quality edible fish oil [33,34].

### 2.3. Fatty Acid Composition

The fatty acid profile of oil obtained after enzymatic hydrolysis from samples subjected to HPP and the control at day 0 of storage is presented in Table 1. There was a significant difference in the content of saturated fatty acids (SFAs), monounsaturated fatty acids (MUFAs) and polyunsaturated fatty acids (PUFAs) between the samples pretreated by HP and the control. The fatty acids C18:1 n-9, C18:2 (n-6), and C16:0 were the most abundant across all samples, with their contents approximately at 39%, 14%, and 10%, respectively. However, about half of the detected fatty acids, including C14:0, C16:1 (n-7), C18:1 (n-7), C18:4 (n-3), C20:0, C20:1 (n-9), C20:4 (n-6), and C22:1 (n-9), did not exhibit a significant difference in relation to the applied pretreatment. Furthermore, the levels of both C20:5 (n-3) (EPA) and C22:6 (n-3) (DHA) were consistent across all samples at approximately 3%. Despite the significant differences detected in the content of fatty acids among the oil samples, these changes were not substantial enough to be attributed to the HP pretreatment. This observation aligns with the findings reported by Yagiz, et al. [35] regarding the impact of HPP and cooking on the quality of Atlantic salmon. Moreover, although unsaturated fatty acids are susceptible to oxidation, the fatty acid profile did not reflect the changes observed in TBARSs (Figure 2). This result is in accordance with the research conducted by Kvangarsnes, et al. [36], where oxidation did not significantly alter the fatty acid profile of oil recovered from rainbow trout heads. Therefore, the observed differences in fatty acid content among the samples could possibly be due to variations in the fatty acid composition of the initial raw material.

### 2.4. Color Measurements (Yellowness)

No significant difference was detected in yellowness between the oil derived from samples pretreated with HPP and the control immediately after production and after 2 and 4 weeks of storage (Table 2). However, after 8 weeks of storage, a significant difference in color was detected among the samples. Furthermore, the oil extracted from the sample subjected to pretreatment at 600 MPa × 4 min exhibited a notable change in color over the storage period. The observed color change is correlated (r = 0.931) with the levels of CT, suggesting that this color variation could be attributed to the formation of compounds that induce a yellow hue following the degradation of primary lipid oxidation products [37].

## 3. Materials and Methods

### 3.1. Preparation of Samples

Fresh Atlantic salmon and rainbow trout rest raw material, including heads, tails, skin, bones, and trimmings, were minced in a ratio of 1:1 *w*/*w*. This same raw material was previously utilized in a study by Kotsoni, Daukšas, Aas, Rustad, Tiwari and Cropotova [21] and underwent HPP prior to enzymatic hydrolysis, as detailed in their research. The HPP was conducted at pressures of 200, 400, and 600 MPa, with hold times of 4 and 8 min. To optimize the quality of the final product, the hydrolysis process was conducted away from direct sunlight and flushed by nitrogen. Furthermore, chamomile and oregano extracts, prepared as described by Kotsoni, Daukšas, Aas, Rustad, Tiwari and Cropotova [21], served as natural antioxidants in a 1:1 ratio and were introduced in the hydrolysis vessel at a concentration of 1.5 mL/kg of raw material. A portion of the oil fraction obtained after enzymatic hydrolysis from the previous study by Kotsoni, Daukšas, Aas, Rustad, Tiwari and Cropotova [21] was set aside for a storage period of 8 weeks to assess changes in quality. The storage intervals were 0 days, 2 weeks, 4 weeks, and 8 weeks after production. During this period, the samples were stored in a cold room at a temperature of 6 ± 2 °C. After each storage period, the samples were stored at −80 °C until further analysis.

### 3.2. Lipid Oxidation Parameters

Primary lipid oxidation products were assessed by the determination of the peroxide value (PV) and conjugated systems: dienes (CD), trienes (CT), and tetraenes (CTr). Secondary lipid oxidation products were evaluated by determining thiobarbituric acid reactive substances (TBARSs) and Schiff bases (SBs). 

The PV was quantified, according to the AOCS Official Method Cd 8b-90 [38], using an automatic titrator (TitroLine^®^ 7800, Xylem Analytics, Mainz, Germany) coupled with a platinum electrode (Pt 62). The results were expressed as mean ± SD (n = 2) in meq O_2_/kg oil. 

Conjugated systems were evaluated by employing a modified version of the methods described in [37]. The results were expressed as mean ± SD (n = 5) CD, CT, and CTr values in mL/mg oil.

TBARSs were measured based on the method described by Ke and Woyewoda [39], with some modifications. A volume of 200 μL of oil, suitably diluted with chloroform, was weighed in a glass centrifuge tube (10 mL, KIMAX, Fisher Scientific, Milpitas, CA, USA). Then, 5 mL of 2-thiobarbituric acid (TBA) working solution, prepared by mixing 180 mL TBA stock solution, 120 mL chloroform, 15 mL sodium sulfite solution, and 9.45 mL butylated hydroxytoluene (3% BHT in ethanol), were added to the tube, which was further mixed on a whirl mixer for 10–15 s. Subsequently, the tubes were incubated in boiling water bath for 45 min followed by cooling down in cold water. Further, 2.5 mL of 0.28 M trichloroacetic acid (TCA) was inserted in each tube, followed by inversion and centrifugation at 900× *g* for 7 min at 4 °C. The absorbance of the obtained pink phase was measured at 538 nm using a SpectraMax i3x Multi-Mode Plate Reader (Molecular Devices, LLC., San Jose, CA, USA). The results were expressed as mean ± SD (n = 2) in μmol malondialdehyde (MDA)/g oil.

The determination of SBs was carried out in accordance with the procedure outlined by Cropotova, et al. [40], with some modifications. About 0.1 g of oil (±0.01 g) was appropriately diluted with 2,2,4-trimethylpentane, and subsequently, measured spectroflourimetrically using a SpectraMax i3x Multi-Mode Plate Reader (Molecular Devices, LLC., San Jose, CA, USA) at 360 nm excitation and 430 nm emission wavelengths. The results were expressed as mean ± SD (n = 3) in mL/kg oil.

### 3.3. Determination of Free Fatty Acids (FFAs)

The determination of FFAs in the oils was based on the method proposed by Bernárdez, et al. [41] with modifications. Approximately 0.1 g of oil was weighed in a glass centrifuge tube (10 mL, KIMAX, Fisher Scientific, USA) before adding 5 mL of 2,2,4-trimethylpentane and mixing. Then, 1 mL of 5% cupric acetate-pyridine aqueous reagent was introduced into the tubes followed by mixing for 30 s and centrifugation at 2000× *g* for 5 min. The resulting upper layer was measured at 715 nm using a SpectraMax i3x Multi-Mode Plate Reader (Molecular Devices, LLC., San Jose, CA, USA). The results were expressed as mean ± SD (n = 3) % of oleic acid.

### 3.4. Fatty Acid Composition

The fatty acid composition was determined following the procedure outlined in [38]. Approximately 25 mg of oil was dissolved in 1.5 mL of 0.5 N NaOH under heat at 100 °C for 5 min. After cooling, the mixture was esterified by adding 2 mL of BF_3_/methanol, followed by heating at 100 °C for 30 min. Upon cooling, 1 mL of isooctane was added, and the tubes were vortexed. Subsequently, 5 mL of saturated NaCl solution was added. The resulting isooctane layer was transferred into vials and analyzed further by gas chromatography (GC). The gas chromatograph (Perken Elmer Autosystem XL) was equipped with a CB-WAX 52CB capillary column (25 m × 0.25 mm) and a flame ionization detector. The injector and detector temperatures were 220 °C and 250 °C, respectively. The oven temperature was 90 °C and held for 1 min. Then, the temperature was raised to 150 °C at a rate of 45 °C/min, followed by a further increase to 220 °C at a rate of 3.5 °C/min, where it was held for 2 min. Hydrogen was used as the gas carrier. The results were expressed as mean ± SD (n = 3) % area fatty acids in GC.

### 3.5. Color Measurement

Color measurement of the oil was performed using a Minolta CR-400 Chroma Meter (Konica-Minolta, Osaka, Japan) equipped with a nozzle suitable for direct contact with liquids. A white plate was used as a reference to calibrate the instrument prior to the analysis. Yellowness (b*-value) was expressed as mean ± SD (n = 3) according to the Commission Internationale de l’Éclairage Lab scale.

### 3.6. Statistical Analysis

The effects of treatments on the quality of the obtained fish oil was evaluated by ANOVA. A significance level of *p* < 0.05 was established to assess the statistical significance of the observed differences between treatment groups and the control. Post hoc analysis was performed using Tukey’s honestly significant difference (HSD) test. Additionally, multiple regression analysis was used to study the effect of HP pretreatment on lipid oxidation parameters. Pearson’s correlation coefficient was also applied to evaluate the relationship between two variables. The statistical analysis was conducted using the SigmaPlot software, version 15 (Systat Software Inc., San Jose, CA, USA).

## 4. Conclusions

The HP pretreatment significantly influenced various parameters associated with lipid oxidation, free fatty acid content, and color stability of the extracted oil during storage. The current research confirmed previous studies indicating that HPP can induce lipid oxidation. Specifically, the results demonstrated that higher pressures and longer durations of HPP were correlated with increased lipid oxidation during storage, as indicated by the elevated levels of PV, CD, CT, CTr, and TBARS values. Despite the utilization of antioxidants and nitrogen flushing, oils subjected to moderate (400 MPa) and high pressure (600 MPa) did not maintain oxidative stability during storage, showing that antioxidants had a small effect in limiting lipid oxidation. In contrast, the oil obtained from the sample pretreated at 200 MPa × 4 min exhibited stability comparable to or higher than that of the control. Prolonged HP pretreatment appeared to reduce FFA content in the oil, potentially enhancing stability by inactivating lipases and preventing the generation of FFAs during storage. Although variations in fatty acid composition were observed among the oil samples, these differences were substantial enough to be attributed to HPP. Furthermore, observed color changes in the oil during storage were linked to lipid oxidation products, suggesting a potential impact of HPP on the sensory properties of fish oil. Future research is necessary to optimize HPP parameters and to identify effective antioxidants that may enhance the oxidative stability of the oil during storage.

## Figures and Tables

**Table 1:** Fatty acid composition in oil derived from enzymatic hydrolysis on samples consisting of a mixture of rainbow trout and Atlantic salmon rest raw material pretreated by HP and on the control at day 0 of storage, expressed as % area fatty acids in GC (mean ± SD, n = 3).

Fatty Acids	Control	200 MPa		400 MPa		600 MPa	
		4 min	8 min	4 min	8 min	4 min	8 min
C14:0	2.82 ± 0.01 ^a^	2.87 ± 0.05 ^a^	2.91 ± 0.00 ^a^	2.86 ± 0.01 ^a^	2.85 ± 0.02 ^a^	2.91 ± 0.07 ^a^	2.92 ± 0.01 ^a^
C16:0	10.77 ± 0.02 ^a^	11.14 ± 0.04 ^b^	11.32 ± 0.02 ^c^	11.03 ± 0.03 ^bd^	11.00 ± 0.04 ^d^	11.12 ± 0.06 ^b^	11.39 ± 0.05 ^c^
C16:1 (n-7)	3.23 ± 0.05 ^a^	3.25 ± 0.01 ^a^	3.27 ± 0.01 ^a^	3.22 ± 0.02 ^a^	3.22 ± 0.04 ^a^	3.22 ± 0.03 ^a^	3.27 ± 0.02 ^a^
C18:0	3.57 ± 0.00 ^a^	3.72 ± 0.04 ^bc^	3.78 ± 0.01 ^cd^	3.76 ± 0.01 ^bd^	3.70 ± 0.03 ^b^	3.71 ± 0.01 ^b^	3.82 ± 0.03 ^d^
C18:1 (n-9)	39.94 ± 0.02 ^a^	39.61 ± 0.15 ^bc^	39.63 ± 0.05 ^bc^	39.67 ± 0.03 ^ab^	39.88 ± 0.03 ^ab^	39.74 ± 0.10 ^ab^	39.43 ± 0.06 ^c^
C18:1 (n-7)	3.37 ± 0.01 ^a^	3.35 ± 0.02 ^a^	3.36 ± 0.00 ^a^	3.33 ± 0.01 ^a^	3.33 ± 0.00 ^a^	3.35 ± 0.02 ^a^	3.37 ± 0.04 ^a^
C18:2 (n-6)	14.28 ± 0.02 ^a^	14.09 ± 0.05 ^bd^	13.96 ± 0.02 ^c^	14.06 ± 0.02 ^b^	14.18 ± 0.02 ^d^	14.10 ± 0.05 ^bd^	13.90 ± 0.00 ^c^
C18:3 (n-3)	7.07 ± 0.01 ^ac^	6.98 ± 0.03 ^ab^	6.91 ± 0.03 ^b^	6.95 ± 0.04 ^b^	7.09 ± 0.04 ^c^	6.97 ± 0.02 ^ab^	6.93 ± 0.03 ^b^
C18:4 (n-3)	0.81 ± 0.01 ^a^	0.82 ± 0.02 ^a^	0.82 ± 0.01 ^a^	0.82 ± 0.01 ^a^	0.79 ± 0.03 ^a^	0.79 ± 0.01 ^a^	0.82 ± 0.01 ^a^
C20:0	0.50 ± 0.00 ^a^	0.52 ± 0.01 ^a^	0.51 ± 0.01 ^a^	0.51 ± 0.02 ^a^	0.53 ± 0.02 ^a^	0.52 ± 0.02 ^a^	0.53 ± 0.00 ^a^
C20:1 (n-9)	3.56 ± 0.10 ^a^	3.52 ± 0.09 ^a^	3.52 ± 0.07 ^a^	3.55 ± 0.10 ^a^	3.39 ± 0.03 ^a^	3.49 ± 0.07 ^a^	3.46 ± 0.01 ^a^
C20:3 (n-3)	0.59 ± 0.01 ^a^	0.57 ± 0.02 ^ac^	0.52 ± 0.01 ^b^	0.56 ± 0.02 ^abc^	0.55 ± 0.02 ^abc^	0.59 ± 0.02 ^a^	0.53 ± 0.01 ^cb^
C20:4 (n-6)	0.20 ± 0.01 ^a^	0.18 ± 0.01 ^a^	0.21 ± 0.02 ^a^	0.21 ± 0.02 ^a^	0.19 ± 0.01 ^a^	0.21 ± 0.03 ^a^	0.22 ± 0.01 ^a^
C20:5 (n-3)	2.97 ± 0.01 ^ab^	2.95 ± 0.03 ^ab^	2.90 ± 0.01 ^a^	3.03 ± 0.01 ^b^	3.04 ± 0.03 ^b^	2.91 ± 0.01 ^a^	2.91 ± 0.03 ^a^
C22:0	0.23 ± 0.01 ^ab^	0.23 ± 0.01 ^ab^	0.24 ± 0.01 ^ab^	0.22 ± 0.01 ^ab^	0.20 ± 0.03 ^b^	0.24 ± 0.01 ^a^	0.25 ± 0.01 ^a^
C22:1 (n-11)	1.56 ± 0.02 ^a^	1.60 ± 0.02 ^ab^	1.60 ± 0.03 ^ab^	1.62 ± 0.02 ^ab^	1.59 ± 0.02 ^a^	1.59 ± 0.02 ^ab^	1.67 ± 0.03 ^b^
C22:1 (n-9)	0.40 ± 0.02 ^a^	0.42 ± 0.02 ^a^	0.41 ± 0.01 ^a^	0.41 ± 0.01 ^a^	0.43 ± 0.02 ^a^	0.41 ± 0.01 ^a^	0.43 ± 0.01 ^a^
C22:5	1.07 ± 0.00 ^a^	1.06 ± 0.03 ^a^	1.05 ± 0.02 ^a^	1.12 ± 0.01 ^b^	1.08 ± 0.01 ^ab^	1.09 ± 0.03 ^ab^	1.08 ± 0.01 ^ab^
C22:6 (n-3)	3.05 ± 0.02 ^ab^	3.12 ± 0.06 ^a^	3.10 ± 0.04 ^a^	3.07 ± 0.02 ^ab^	2.96 ± 0.04 ^b^	3.04 ± 0.04 ^ab^	3.07 ± 0.01 ^ab^
SFAs ^1^	17.89 ± 0.01 ^a^	18.47 ± 0.02 ^b^	18.75 ± 0.01 ^c^	18.38 ± 0.01 ^bd^	18.28 ± 0.02 ^d^	18.50 ± 0.03 ^b^	18.92 ± 0.03 ^e^
MUFAs ^2^	52.06 ± 0.06 ^a^	51.75 ± 0.06 ^ab^	51.79 ± 0.03 ^ab^	51.81 ± 0.04 ^ab^	51.84 ± 0.02 ^ab^	51.81 ± 0.04 ^ab^	51.63 ± 0.04 ^b^
PUFAs ^3^	30.04 ± 0.02 ^ac^	29.78 ± 0.03 ^ac^	29.46 ± 0.02 ^b^	29.82 ± 0.02 ^ac^	29.88 ± 0.02 ^ac^	29.69 ± 0.03 ^cb^	29.45 ± 0.02 ^b^

Across various treatment groups, values labeled with different lowercase letters indicate significant differences. ^1^ SFAs: saturated fatty acids; ^2^ MUFAs: monounsaturated fatty acids; ^3^ PUFAs: polyunsaturated fatty acids.

**Table 2:** Evaluation of yellowness (b*-values) in oil extracted after enzymatic hydrolysis of a mixture of rainbow trout and Atlantic salmon rest raw material, pretreated by HP, and the control during storage (mean ± SD, n = 3).

HP Pretreatment	Yellowness (b*)			
	0 Days	2 Weeks	4 Weeks	8 Weeks
Control	19.4 ± 2.0 ^Aa^	22 ± 1.3 ^Aa^	18.3 ± 0.7 ^Aa^	19.3 ± 0.8 ^Aab^
200 MPa × 4 min	22.2 ± 2.4 ^Aa^	19.6 ± 1.2 ^Aa^	18.7 ± 0.4 ^Aa^	17.6 ± 0.9 ^Aa^
200 MPa × 8 min	19.8 ± 0.5 ^Aa^	22.5 ± 1.2 ^Aa^	19.5 ± 1.8 ^Aa^	17.4 ± 0.4 ^Aa^
400 MPa × 4 min	21.3 ± 2.1 ^Aa^	21.1 ± 0.6 ^Aa^	19.5 ± 0.2 ^Aa^	20.3 ± 0.3 ^Aab^
400 MPa × 8 min	22.4 ± 1.3 ^Aa^	20 ± 3.4 ^Aa^	18.9 ± 0.9 ^Aa^	21.1 ± 1.6 ^Ab^
600 MPa × 4 min	20.3 ± 0.9 ^ABa^	21.7 ± 0.8 ^Aa^	21.2 ± 0.3 ^Aa^	18.7 ± 0.0 ^Bab^
600 MPa × 8 min	22 ± 0.6 ^Aa^	21.5 ± 1.6 ^Aa^	19.9 ± 1.1 ^Aa^	19.2 ± 0.3 ^Aab^

In each treatment group, values marked with distinct uppercase letters denote a significant difference. Across various treatment groups, values labeled with different lowercase letters indicate significant differences.

## Data Availability

Data are contained within the article.
